# Characterisation of the bacteriomes harboured by major wireworm pest species in the Canadian Prairies

**DOI:** 10.1111/imb.12962

**Published:** 2024-10-09

**Authors:** Ivan Drahun, Keagan Morrison, Elise A. Poole, Willem G. van Herk, Bryan J. Cassone

**Affiliations:** ^1^ Department of Biology Brandon University Brandon Manitoba Canada; ^2^ Agassiz Research and Development Centre Agriculture and Agri‐Food Canada Agassiz British Columbia Canada

**Keywords:** agricultural pests, bacteriome, metagenomics, wireworms

## Abstract

Nearly all insects harbour bacterial communities that can have a profound effect on their life history, including regulating and shaping host metabolism, development, immunity and fitness. The bacteriomes of several coleopterans have been described; however, very little has been reported for wireworms. These long‐lived larvae of click beetles (Coleoptera: Elateridae) are major agricultural pests of a variety of crops grown in the Canadian Prairies. Consequently, the goal of this study was to characterise the bacteriomes of five of the most significant pest species within the region: *Limonius californicus*, *Hypnoidus abbreviatus*, *H. bicolor*, *Aeolus mellillus* and *Dalopius* spp. To do this, we collected larvae from southern Manitoba fields (pre‐seeding) and carried out 16S rRNA sequencing on individual specimens. Our results indicate wireworms have diverse and taxon‐rich bacterial communities, with over 400 genera identified predominately from the phyla Proteobacteria, Actinobacteriota, Bacteroidota and Firmicutes. However, each species had nine or fewer genera comprising >80% of their bacteriome. Network analyses revealed some community structuring consistent among species, which may culminate in shaping/regulating host biology. Moreover, the microbial signatures were influenced by both ontogeny (early vs. late stage larvae) and reproductive strategy (sexual vs. parthenogenetic), with a myriad of other factors likely contributing to bacterial diversity that are impossible to resolve from our study. Overall, this metagenomics study represents the first to characterise the bacteriomes of wireworms in the Canadian Prairies and the findings could assist in the development of sustainable management strategies for these important agricultural pests.

## Graphical abstract

- The bacteriomes of five of the most important agricultural pests in the Canadian Prairies were characterised.
- Wireworms have diverse and taxon‐rich bacterial communities, with different species having a distinct composition and a small number of genera predominating.
- The wireworm bacteriomes are influenced by development and reproductive strategy, among several other putative factors.

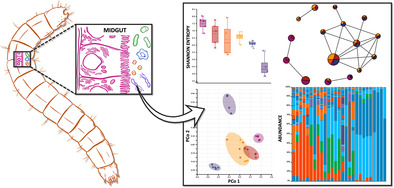

## INTRODUCTION

Bacterial symbionts are harboured to some extent by virtually all insects, with such host–microbe associations occurring on a continuum from detrimental to beneficial (Gedling et al., 2018; Paddock et al., 2022; Wielkopolan et al., 2021). Pathogens are capable of impacting insect fitness and causing mortality under certain circumstances (Caccia et al., 2016; Mason et al., 2018; Sisterson, 2009). In turn, some symbionts can regulate/shape host development, metabolism and immunity, as well as play key roles in insect ecology and evolution (Douglas, 2016; Frago et al., 2012; Janson et al., 2008; McFall‐Ngai et al., 2013; Sudakaran et al., 2017). For herbivorous insects, their bacterial assemblages have been shown to mediate host plant preferences (De Vries et al., 2006; He et al., 2022; Lu et al., 2016; Xu et al., 2016), aid in digestion (Barbosa et al., 2020; Dantur et al., 2015) and degrade/metabolise plant toxins (Hammer & Bowers, 2015; Mason, 2020; Zhang et al., 2022). A myriad of factors can change/alter an insect's ‘bacteriome’, including host diet, ontogeny and environment (Colman et al., 2012; Gohl et al., 2022; Magoga et al., 2022; Nobles & Jackson, 2020). Consequently, bacteriomes have been shown to vary among different insect species, within populations of the same species and even among individuals within populations (Lange et al., 2023).

Coleoptera is regarded as the most abundant and diversified order of insects, comprising more than 360,000 species (Frago et al., 2020). To date, the bacteriomes of several beetle species have been characterised (Adams et al., 2013; Ebert et al., 2021; Estes et al., 2013; Magagnoli et al., 2022; Moldovan et al., 2023; Scully et al., 2014), including the larval stages of the dung beetle (Suárez‐Moo et al., 2020), cereal leaf beetle (Wielkopolan et al., 2021), green June beetle (Kucuk et al., 2023) and various xylophagous species (Mohammed et al., 2018). Most of these studies have focused on the microbial communities inhabiting the insect's intestinal tract, though salivary gland oral secretions have also been examined in leaf feeding species (Chung et al., 2013; Gedling et al., 2018). For these herbivorous coleopterans, tripartite interactions among insect, host plants and their bacteriome are fundamental to their success (Wielkopolan & Obrępalska‐Stęplowska, 2016). While we are still in the early stages of understanding the diversity and dynamics of the microorganisms they harbour, Firmicutes, Actinobacteris, Bacterioidota and Proteobacteria tend to be the most represented bacterial phyla (Falqueto et al., 2022; Gedling et al., 2018; Jones et al., 2013).

Wireworms, the long‐lived subterranean larval stages of click beetles (Coleoptera: Elateridae), are regarded as significant pests globally (Drahun et al., 2023; Labrie et al., 2020; Vernon et al., 2016). While the adult beetles do not pose a serious threat as agricultural pests, their larval stages feed on various crops, including legumes, cereal grains and root vegetables (Rashed & van Herk, 2024; Traugott et al., 2015). Wireworm feeding activities can damage plants (e.g., cosmetic injury, stunting, wilting and death) and result in bare patches and considerably reduced crop yields in heavily infested fields (Barsics et al., 2013; Parker & Howard, 2002; Ritter & Richter, 2013; van Herk & Vernon, 2014). Before their phase‐out in most countries in the early 2000s, wireworms were effectively controlled by broad‐spectrum insecticides such as lindane and aldrin. Current management tactics are usually part of integrated strategies that temporarily abate larval feeding to enable early season crop establishment (e.g., neonicotinoid seed treatments), reduce wireworm numbers to an extent (e.g., crop rotation, cultivation, entomopathogenic microorganisms, broflanilide) or target the adult beetles to indirectly decrease larval numbers (e.g., mass trapping, attract‐and‐kill, mating disruption) (Brandl et al., 2017; Labrie et al., 2020; Ritter & Richter, 2013; van Herk et al., 2022; van Herk et al., 2023; van Herk, Vernon, Goudis, & Mitchell, 2021; Vernon et al., 2016; Vernon & van Herk, 2022). Longer term, studies aimed at better understanding the bacteriomes of wireworms and their associated roles with insect physiology and taxonomy would enrich our understanding of insect–microbe interactions and could also facilitate the development of novel and sustainable pest control tools and strategies (Kabaluk et al., 2007; Kleespies et al., 2012; Lacey et al., 2007; Rupawate et al., 2023; Zhang et al., 2023).

Several economically important wireworm pest species are endemic to the Canadian Prairie provinces of Manitoba, Saskatchewan and Alberta. Given the terricolous behaviours of wireworms, most field surveys of species composition and abundance utilise indirect sampling approaches (e.g., bait trapping) that largely reflect larval activity at the time of collection (Drahun et al., 2021). Moreover, there is evidence that some species become active earlier/later in the growing season (Drahun et al., 2023). In early spring, *Hypnoidus bicolor* is far and away the most commonly found species in Manitoba (>90% of samples) and to a lesser extent in Saskatchewan (>40%) and Alberta (>30%) (Drahun et al., 2021; van Herk, Vernon, Labun, et al., 2021). A member of the same genus, *H. abbreviatus* is the most abundant pest species in Quebec (Saguez et al., 2017) but comprises <10% of samples in the Prairies (Drahun et al., 2021; van Herk, Vernon, Labun, et al., 2021). *Limonius californicus* is largely constrained to irrigated areas of the southern prairies and tends to become more active as the growing season progresses into early summer (Drahun et al., 2023). *Selatosomus aeripennis destructor* was historically the most ubiquitous pest species in Manitoba (Glen et al., 1943), but its populations are now largely restricted to Saskatchewan and Alberta (van Herk, Vernon, Labun, et al., 2021). Other pest species of note present in the Prairies albeit at relatively low abundances include *Aeolus mellillus*, *Agriotes mancus* and *Dalopius* spp. (Drahun et al., 2021; van Herk, Vernon, Labun, et al., 2021).

Little information is presently available regarding the composition of wireworm bacteriomes. The earliest documentation of bacteria harboured by larvae were eight Eubacteriineae isolated from *Melanotus caudex* (Yoshida & Yoshii, 1959). In the 1970s, *Pseudomonas aeruginosa* was identified in *L. californicus* larvae (Zacharuk, 1973a; Zacharuk, 1973b; Zacharuk, 1974). Subsequent studies isolated various bacterial flora from *Agriotes* spp., largely focusing on pathogenic species (Danismazoglu et al., 2012; Kabaluka et al., 2017; Kleespies et al., 2012; Leclerque et al., 2011; Schuster et al., 2013). Lacey et al. (2007) used 16S rDNA sequencing to identify 86 bacterial isolates from the guts of the Pacific Coast wireworm, *L. canus*, which largely consisted of *Pseudomonas* and *Bacillus*. Because all of these previous studies selectively isolated bacteria from the intestines or other tissues, virtually nothing is known about the natural composition and structure of the flora within the larvae. Therefore, the objective of our study was to characterise and contrast the bacterial communities associated with five notable Canadian Prairie pest species: *L. californicus*, *H. abbreviatus*, *H. bicolor*, *Ae. mellillus* and *Dalopius* spp. To do this, we sampled the larvae from Manitoba crop fields and subjected individuals to 16S amplicon sequencing and metagenomics analysis. Overall, this type of research could open new avenues for sustainable and environmentally friendly management of these economically significant coleopterans, including the development of microbial insecticides and plant‐incorporated protectants and semiochemicals (Qadri et al., 2020).

## MATERIALS AND METHODS

### Wireworm sampling

In 2021, two fields located in southwestern Manitoba with a recent history of wireworm infestations were selected for collections: ED: 49.6507, −99.7052 and MW: 49.1664, −99.3190. We sampled the fields simultaneously in mid‐April (pre‐seed) using bait traps that consisted of 30 mL untreated and sterilised spring wheat seed (*Triticum* spp., cultivar Carberry) sandwiched between two layers of coarse vermiculite (Grade 4) in a green DILLEN resin planter pot (8.89 × 10.16 cm) (Vernon et al., 2009; Vernon et al., 2013). Each trap was saturated to stimulate germination and then placed into 15 cm holes organised into 4 linear transects (*n* = 6 traps per transect) along the field peripheries. We then covered each trap with 2.5 cm of soil, placed an upside‐down plastic saucer (20 cm diameter) on top (to protect against rainfall and augment sunlight absorption) and marked the location using a stake flag and GPS tag. After 14 days in the ground, we recovered the traps and manually extracted the wireworms by carefully sifting through each trap three times using a combination of sieves: 2.5, 2 and 1.25 mm in pore size. Where possible, each larva was identified to the species using reference keys based on a combination of morphological features (Brooks, 1961; Etzler, 2013; Glen et al., 1943). Wireworm were surface sterilised using one drop of Tween® 80 per 10 mL of 0.5% bleach, followed by 0.5% benzalkonium chloride and 70% ethanol (Yunik et al., 2015). We stored the larvae in individual 2 mL tubes at −80°C until DNA isolation.

### DNA extraction and amplicon sequencing

All DNA isolations were carried out on individual wireworms. To do this, we first excised the cuticle of each larva using a new set of sterilised scalpels (size 10 blades) and forceps per specimen. The remaining sample was subjected to gDNA extraction using the One‐4‐All Genomic DNA Miniprep Kit (Bio Basic, Markham, ON). In addition, gDNA from three negative control samples (each containing lysis buffer devoid of wireworm tissues) were extracted and processed identically to the experimental samples. We evaluated the quality and quantity of DNA using the Nanophotometer NP80 (Implen Inc., Westlake Village, CA). Amplification of the V4 region of the 16S rRNA gene was done in 40 μL reactions using Platinum SuperFi PCR Master Mix (Invitrogen, Waltham, MA) and the following adapter‐tagged primer set: 515F: *ACACTCTTTCCCTACACGACGCTCTTCCGATCT*GTGCCAGCMGCCGCGGTAA and 806R: *GTGACTGGAGTTCAGACGTGTGCTCTTCCGATCT*GGACTACHVGGGTWTCTAAT (Caporaso et al. 2011). These adaptors serve as barcodes, which are pertinent for downstream sample identification and allows for multiplexing. Each PCR run included a no template control (*n* = 2), which was sequenced concurrently with the extraction negative controls. PCRs were done in a Biometra TOne thermal cycler (Analytik Jena AG) using the following thermal regime: 98°C for 30 s, followed by 35 cycles of denaturation at 98°C for 5 s, annealing at 65°C for 20 s, elongation at 72°C for 15 s and a final elongation step at 72°C for 5 min (Boelsen et al., 2019). We sent the adapter‐tagged amplicons (product size of ~300 bp) to the Génome Québec Innovation Centre (McGill University, Montreal, QC, Canada) for library barcoding and quality assessment using the BioAnalyzer 2100 (Agilent Technologies, Santa Clara, CA) and sequencing on the Illumina MiSeq platform in paired‐end 250 bp fashion. The raw sequence reads can be retrieved from the NCBI short sequence read archive (SRA) under the accession number PRJNA1098768.

### Data analysis

We analysed the amplicon sequencing reads using CLC Genomics Workbench 23.0.1 and CLC Microbial Genomics Module 23.0.1 software. Raw reads were imported into the software, trimmed for quality using default parameters (Quality limit = 0.05 and Ambiguous limit = 2), and the adapter and chimeric were also removed. Operational taxonomic unit (OTU) clustering was done de novo at ≥99% sequence similarity (default settings herein) using reference‐based OTU picking against the SILVA SSU (release 138.1) database (Quast et al., 2013). This included the alignment of OTUs using MUSCLE (non‐merged reads were omitted) and the construction of Maximum Likelihood Phylogeny using default parameters. We filtered the resulting abundance tables to remove OTUs present in <0.05% of the total reads. The de novo OTUs without taxonomy were contrasted against the nr database and UNITE database using their respective BLASTn tool and default setting to assign taxonomy whenever possible.

Statistical analyses were carried out to explore differences in the bacterial communities across wireworm pest species using default parameters (unless indicated otherwise) and the work flows provided in the CLC microbial module. We estimated alpha diversity (Shannon Entropy) at the OTU level, with rarefication set to minimum/maximum depths of 1/8164 to ensure all sample curves reached a horizontal asymptote. Shannon Entropy values were retrieved for the highest rarefication level by sampling 20 unique depths at regular intervals, with 100 subsamples of the data at each point (without replacement). Significance was assigned to alpha diversity using the Kruskal–Wallis test and Dunn's post hoc test (*p* < 0.05), performed in the ‘MultNonParam’ package (Kolassa & Seifu, 2013) in R (R Core Team, 2021). Beta diversity (Bray–Curtis) was also estimated at the OTU level, with these indices used to (1) construct principal coordinate analysis (PCoA) plots and (2) perform permutational multivariate analysis of variance (PERMANOVA) with Jaccard distance and a *p* < 0.05 threshold for significance. Taxonomic profiling was done using the QMI‐PTDB curated reference database and a minimum seed length of 30.

We used the differential abundance analysis (DAA) tool in CLC to carry out pairwise comparisons between species at the genus and phylum levels. For this, Generalised Linear Models (GLMs) are performed on a given feature (e.g., a phylum) and significance is determined using the Wald test. Only taxon with a combined abundance of ≥100 reads and/or ≥3 replicates with reads (i.e., non‐zero) for at least one species were considered for this analysis. Significance was set to an FDR‐adjusted *p* < 0.05 and log_2_ fold‐change ≥|1.5|. Genus‐level network analyses were also done across species using the Marker Data Profiling tool in MicrobiomeAnalyst (Chong et al., 2020; Dhariwal et al., 2017) and the Spearman's rank correlation feature with a *r* > 0.6 and *p* < 0.05 (default setting herein). This analysis identifies significant associations between bacterial genera based on similarities (i.e., patterns) in relative abundance across treatments (e.g., wireworm species).

## RESULTS

### Wireworm sampling

Larvae representing four wireworm pest species were collected via bait traps and used in our study, with one of the species (*L. californicus*) separated into two size ranges: *H. bicolor* (*n* = 5; 9–10 mm), *H. abbreviatus* (*n* = 5; all 11 mm), *Ae. mellillus* (*n* = 4; 12–13 mm), *L. californicus*‐large (*n* = 4; 20–21 mm) and *L. californicus*‐small (*n* = 4; 8–9 mm). Larger wireworm sizes are indicative of later life stage; thus, we included both early and late stage specimens of this species. In addition, we sampled individuals from the genus *Dalopius* (*n* = 5; 13–14 mm); although the specimens could not be resolved to the species‐level, they were morphologically indistinguishable and are herein referred to as *Dalopius* spp. All wireworms were collected from ED (49.6507, −99.7052) with the exception of *H. abbreviatus* (MW: 49.1664, −99.3190).

### Sequencing and taxonomic analysis

Our study aimed to characterise the bacterial communities harboured by five wireworm pest species endemic to the Canadian Prairies. The bacteriomes presented reflect individual specimens (i.e., not pooled samples) replicated 4 or 5 times per species and sampled from the same agricultural field (with the exception of *H. abbreviatus* being collected from a nearby field). A total of 1,105,540 paired‐end reads were generated (not including the negative controls), and we omitted reads from the dataset that were of low quality, chimeric sequences and/or unable to be resolved to the OTU level (∼38%) (Table S1). After exclusion of these sequences, 16S amplicon analysis of the bacterial communities identified a total 7885 OTUs across the five species (1031–2626 per species). Table 1 shows the taxonomic breakdown for each pest species. The negative controls were largely composed of *Escherichia*–*Shigella*, *Sphingomonas*, *Brevibacterium*, *Anaerococcus*, *Cutibacterium* and uncultured bacterium. Consequently, these genera were omitted from our dataset to mitigate any influence of reagent‐derived contaminants.

**TABLE 1:** Taxonomic breakdown of the bacteria harboured by each wireworm pest species.

Taxonomic level	*Limonius californicus* (S)	*L. californicus* (L)	*Hypnoidus abbreviatus*	*Dalopius* spp.	*Aeolus mellillus*	*H. bicolor*
Kingdom	2	2	2	1	1	2
Phylum	11	12	12	9	5	16
Class	14	19	15	10	6	23
Order	26	36	28	19	8	46
Family	31	45	35	26	11	62
Genus	43	64	56	30	11	105
OTU, total	1228	1961	1883	1031	1065	2626
OTU, database	189	265	188	80	107	410
OTU, de novo	1039	1696	1695	951	958	2216

*Note*: Only taxa represented by >0.05% of reads for a given species are included.

Abbreviation: OTU, operational taxonomic unit.

### The wireworm bacteriome

**FIGURE 1:**
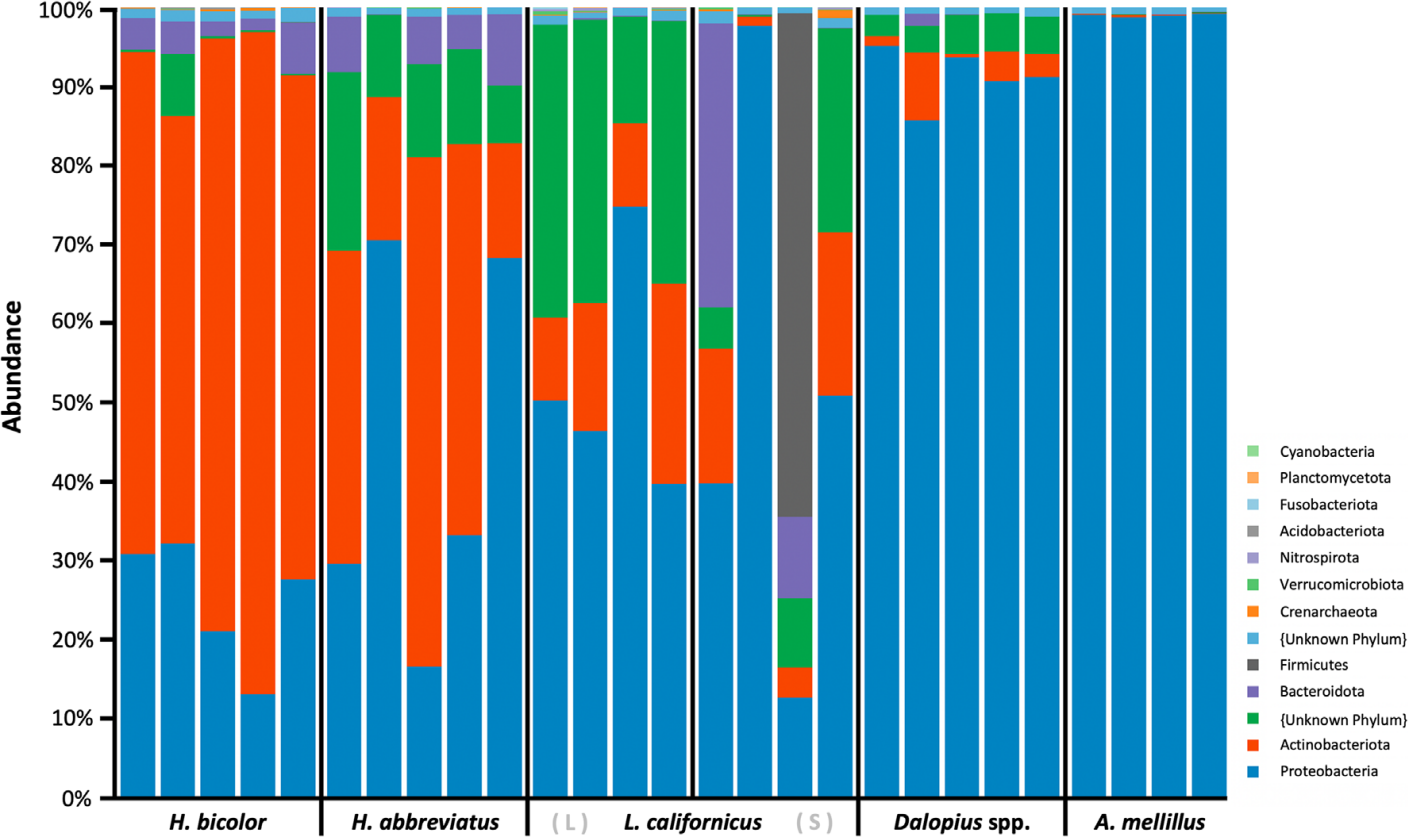
Relative abundances of bacterial phyla identified in five wireworm pest species. Each box plot represents a biological replicate for a given species. A total of 30 phyla were identified across species, of which the 13 most represented are shown.

As a first step, we filtered the bacterial communities to the phylum level to gain a broad understanding of the bacteriome across different species (Figure 1). With the exception of large *L. californicus*, the relative proportions of phyla within each species were relatively consistent across replicates (i.e., individuals). A total of 30 phyla were identified across species, though only 14 had a combined abundance across species of over 100 reads. Proteobacteria dominated the microbial signature for *Ae. mellillus* (99% of species‐specific sequencing reads), *Dalopius* spp. (91%) and to a lesser extent *L. californicus* (55%, both sizes) and *H. abbreviatus* (47%). Actinobacteriota was the most abundant phylum for *H. bicolor* (63%), with Proteobacteria comprising 27% of reads for this species. Actinobacteriota was also well represented in *H. abbreviatus* (34%) and *L. californicus* (8%–15%). Fusobacteriota was the only other phylum identified across all species/replicates, albeit in relatively low numbers (<0.05%). Other notable phyla included Bacteroidota (i.e., third most abundant phylum) and Firmicutes (i.e., found across species including 18% of large *L. californicus*).

### Alpha diversity

**FIGURE 2:**
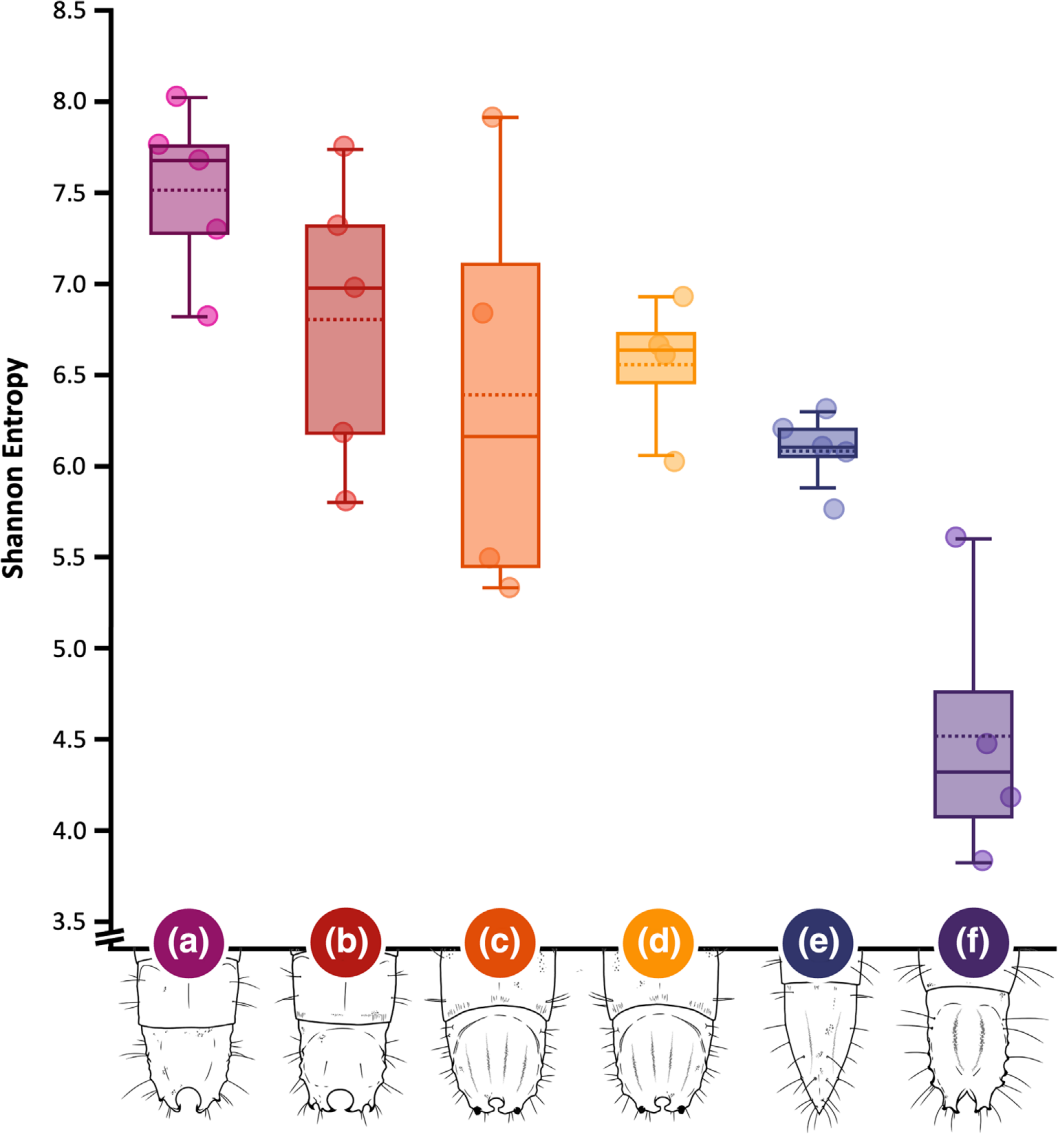
Shannon entropy indices of alpha diversity for the five wireworm pest species: (a) *Hypnoidus bicolor*; (b) *Hypnoidus abbreviatus*; (c) *Limonius californicus* (large); (d) *Limonius californicus* (small); (e) *Dalopius* spp. and (f) *Aeolus mellillus*. The indices are operational taxonomic unit (OTU) based.

To gain insights into how the richness and evenness of bacterial assemblages varied among the wireworm pest species, we measured quantitative OTU‐based indices of alpha diversity, calculated as Shannon Entropy (Figure 2) (Hughes et al., 2001; Lozupone & Knight, 2008). Alpha diversity ranged between 4.5 and 7.5 and was significantly lower in *Ae. mellillus* relative to *H. bicolor* (*z* = 3.6811, *p* < 0.0002), *H. abbreviatus* (*z* = 2.6669, *p* = 0.0077) and small *L. californicus* (*z* = 2.0935, *p* = 0.0363). The only other statistically significant difference was higher alpha diversity in *H. bicolor* than *Dalopius* spp. (*z* = 2.3108, *p* = 0.02085).

### Beta diversity

**FIGURE 3:**
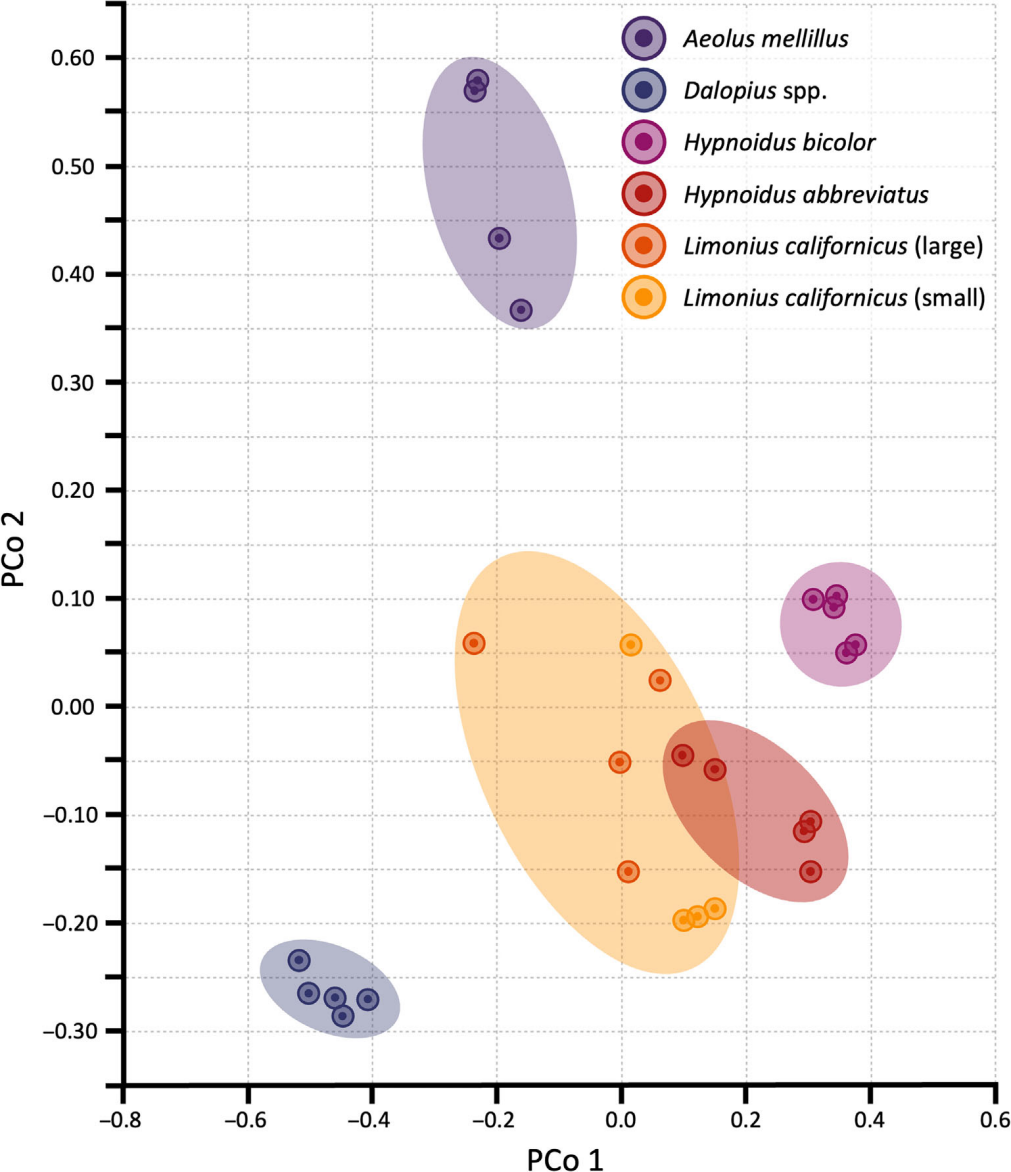
Principal coordinate analyses (PCoA) of bacteria harboured by Canadian Prairie wireworm species. Distinct clustering was observed between species and to a lesser extent developmental stages (*Limonius californicus*).

While alpha diversity quantifies the bacterial richness and evenness within a given wireworm individual/species, beta diversity provides information into how the composition of these communities differs among pest species. We constructed two‐dimensional PCoA plots to explore and visualise the beta diversity distance matrices calculated using Bray–Curtis dissimilarity with independently plotted biological replicates (Figure 3). We observed discrete clustering among wireworm species, indicating that the abundance and structure of their bacterial communities was divergent. The top two and three components accounted for 54% and 72% of variability, respectively. This separation among species was largely supported by PERMANOVA analysis, which revealed significant overall differences among species (*p* = 0.00001), as well as for each pairwise species comparison (*p* < 0.028 for all pairwise contrasts). The only non‐significant comparison was between *Ae. mellillus* and large *L. californicus* (*p* = 0.114). Moreover, the comparison between small and large *L. californicus* was also non‐significant (*p* = 0.057). The non‐significance between *Ae. mellillus* and large *L. californicus* notwithstanding the distinct clustering on the PCoA, may be at least partially attributed to the similar patterns in relative abundance of three predominant bacterial genera (*Pseudomonas*, *Serratia* and *Gammaproteobacteria*).

### Molecular signatures of wireworm pest species

**FIGURE 4:**
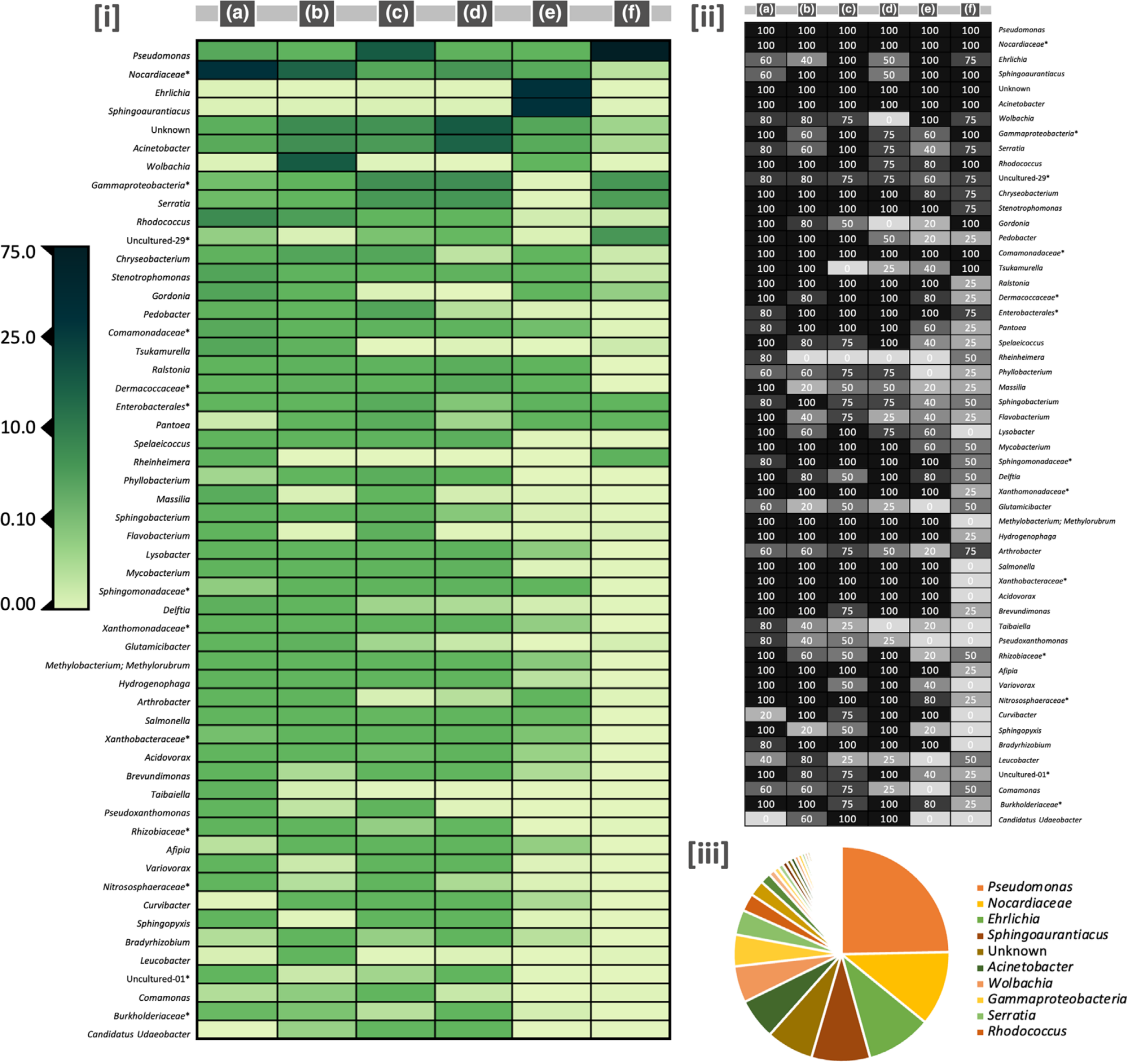
Relative abundances of the most common bacterial genera inhabiting Canadian Prairie wireworm species: (a) *Hypnoidus bicolor*; (b) *Hypnoidus abbreviatus*; (c) *Limonius californicus* (large); (d) *Limonius californicus* (small); (e) *Dalopius* spp. and (f) *Aeolus mellillus*. This figure displays (i) heat map showing the proportion of reads representing each genus for a given species, as indicated by the scale bar; (ii) proportion of biological replicates for each species for which a given bacterial genera was detected and (iii) most represented bacterial genera overall (i.e., across species). Although all genera are provided, the legend only displays the 10 most abundant.

Since wireworm species differed considerably in the composition of their bacterial communities, we next wanted to characterise taxa with the most pronounced influence on the respective larval bacteriomes. While we focus primarily on genus‐level changes due to the greater resolution for biological interpretation, DAA was also performed at the phylum level. In some cases, the bacterial family/order is provided as the genus is unknown. A total of 419 genera were identified across the five species, with 79 meeting our criterion for analysis. Figure 4 displays a heat map of the relative abundances for the most represented genera among the species, and the proportion of biological replicates where a given genus is present. Pairwise DAA statistical differences between larval species are shown in Table S2, whereas Figure S1 displays the relative proportions of the most common genera for each species/replicate. The microbial signatures of all five wireworm species differed extensively from one another. In most cases, the dominant bacterial genera were significantly enriched in larvae of one species relative to the other four, with nearly all (78 of 79) genera showing significant differential abundance in at least one pairwise comparison. We also identified considerable statistical differences between small and large *L. californicus*, albeit not as extensive as most interspecies comparisons.

*Ehrlichia* (45% of species‐specific sequencing reads) and *Sphingoaurantiacus* (40%) dominated the microbial signature of *Dalopius* spp., with both genera found in negligible numbers in the other wireworm species (<0.01%). *Pseudomonas* comprised 74% of the bacteriome of *Ae. mellillus*, with uncultured‐29 (8.7%), Gammaproteobacteria (unknown genus) (8.6%) and *Serratia* (6.8%) also found in relatively high abundance. Nocardiaceae (unknown genus) was the most common bacteria found in *H. bicolor* (42%), with *Rhodococcus* (12%), *Stenotrophomonas* (5%), *Gordonia* (5%) and *Tsukamurella* (4.2%) enriched in the larva compared to other wireworm species. Both Nocardiaceae (23%) and *Rhodococcus* (6.6%) were also well represented in the other *Hypnoidus* wireworm, *H. abbreviatus*. However, *Wolbachia* was the primary bacteria harboured by this species, with 30% of sequencing reads in comparison to <0.15% for larvae of the other four pests. Other notable genera for *H. abbreviatus* included *Acinetobacter* (11%) and *Chryseobacterium* (4.5%). The bacteriomes of both small and large *L. californicus* had relatively high proportions of *Acinetobacter* (S: 23%; L: 7.6%), Gammaproteobacteria (S: 11%; L: 11%), *Serratia* (S: 8.9%; L: 8.2%), Nocardiaceae (S: 9%; L: 4%). However, *Pseudomonas* (32%) was the predominant genus in large larva, with Pedobacter (4.8%) and Chryseobacterium (4.4%) also enriched in comparison to small larva. Despite the extensive differences in bacterial communities across species, only two putative genera were uniquely found in a given wireworm: uncultured‐09 and Rickettsiales (unknown genus) in *Dalopius* spp. In both cases, the bacteria were present in all five biological replicates, albeit a low overall abundance (<0.12% of sequencing reads). Table S3 provides a general overview of the habitat(s) the core bacterial genera (>1000 combined abundance) are commonly associated with.

### Network analysis

**FIGURE 5:**
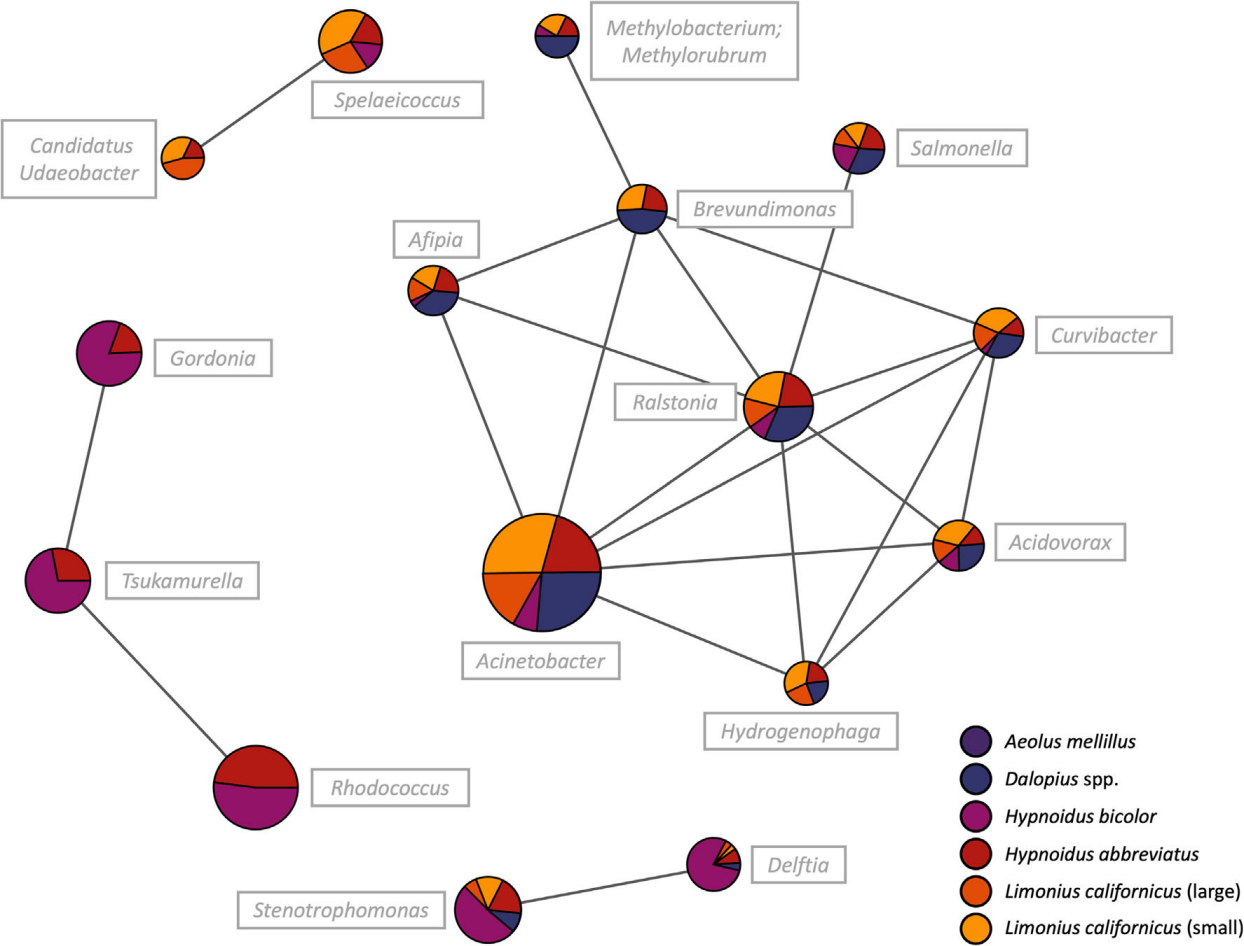
Genus‐level network analyses of the microbiomes of five Canadian Prairie wireworm species. Relationships among genera were derived using Spearman's rank correlation and a threshold for significance of *p* < 0.05 and *r* > 0.6. A total of four networks were identified across species. The size of each pie chart corresponds to combined bacterial abundance and each slice represents a given wireworm species.

Network analyses revealed positive correlations between some of the genera harboured across wireworm species, which were separated into four distinct networks (Figure 5). Three of the networks were comprised of only two or three genera: *Rhodococcus*, *Gordonia* and *Tsukamurella*; *Stenotrophomonas* and *Delftia*; *Spelaeicoccus* and *Candidatus* Udaeobacter. The primary network consisted of positive associations among nine genera: *Acinetobacter*, *Ralstonia*, *Acidovorax*, *Brevundimonas*, *Curvibacter*, *Hydrogenophaga*, *Salmonella*, *Methylobacterium–Methylorubrum* and *Afipia*. Most of the networks reflected a co‐occurrence of all larval species, with the exception being the absence of *Ae. mellillus* from all networks. Moreover, *Rhodococcus*, *Gordonia* and *Tsukamurella* was a network specific to the *Hypnoidus* species.

## DISCUSSION

Wireworms are significant pests to a variety of crops grown in the Canadian Prairies (Catton et al., 2021); however, no studies have previously been undertaken to explore the larval bacteriomes of these terricolous coleopterans. Therefore, our study represents the first efforts to characterise the bacterial communities harboured in five of the most economically important species endemic to this region. To mitigate the potential influence of environment and ontogeny on the bacterial assemblages, we endeavoured to collect wireworms at the same time of year from one location (the exception being *H. abbreviatus* collected from a different field in close proximity) and ensured all biological replicates were of similar size (within 1 mm). It should be noted that the read counts for a given taxon are a good indicator of relative abundance but do not necessarily correlate with overall bacterial densities. This is because genome size cannot be normalised for relative abundance, as this parameter varies between bacterial species and is not available for most taxa. Our findings indicate that the wireworm bacteriome is host species‐specific, although the overall microbial signatures were dominated by a handful of taxa that, in many cases, were present across multiple species. This interspecific divergence is consistent with Mohammed et al. (2018), who compared the bacterial communities of various xylophagous beetle larvae. While ontogeny plays a key role in shaping the coleopteran bacteriome (Chouaia et al., 2019; Suárez‐Moo et al., 2020), our analysis was limited to small (i.e., early life stage) and large (i.e., late life stage) *L. californicus* and, as expected, did not reveal the same magnitude of changes as those among species.

Similar to what is known about most coleopterans (Falqueto et al., 2022; Gedling et al., 2018; Jones et al., 2013; Mohammed et al., 2018), wireworms predominately harboured Proteobacteria and Actinobacteriota, with Bacteroidota and Firmicutes also occurring in relatively high proportions. Bacterial richness and evenness were fairly consistent across species, with the exception being *Ae. mellillus* larvae. This species showed reduced alpha diversity, which may be related to its reproductive strategy. Of the five species of interest, all known populations of *Ae. mellillus* are parthenogenetic, meaning their embryos develop from unfertilized eggs, and all individuals are female (Catton et al., 2021; Glen et al., 1943). Little has been reported in the literature regarding the impact of parthenogenesis on host microbial diversity; however, sex‐biased bacteriomes are common among insects, and this could potentially constrain species richness for clonal species (Muratore et al., 2020; Wan et al., 2020). Indeed, several genera were absent in *Ae. mellillus* in comparison to all of the other wireworm species, including *Chloroflexi*, *Nitrospirota* and *Planctomycetota*. On the other hand, some microorganisms are associated with parthenogenesis in insects, as they alter/interfere with host reproduction to support their persistence in populations (Bisschop et al., 2022; Schön et al., 2009; Stouthamer et al., 1999). This includes members of the genera *Spiroplasma*, *Wolbachia*, *Rickettsia* and *Cardinium* (Fialho & Stevens, 2000; Harumoto & Lemaitre, 2018; Hurst et al., 1999; Hurst & Jiggins, 2000; Katsuma et al., 2022; Leclerque et al., 2011; Schuster et al., 2013; Stouthamer et al., 2019). Of these, only *Wolbachia* was identified in *Ae. mellillus*, but in negligible abundance (0.002%) compared with the bacteriomes of some of the other wireworm species.

Despite the identification of more than 400 bacterial genera across wireworm species, each species had nine or fewer genera comprising >80% of their bacteriome. Most striking were *Ae. mellillus* (4 genera, >98%), *Dalopius* spp. (5 genera, >95%) and *H. abbreviatus* (6 genera, >84%). Moreover, the six most abundant genera were present across all larval species and individual replicates, though each was enriched in only one or two species. Although it must be emphasised that little is known about the symbiotic relationships between coleopterans and their bacteriome, including virtually nothing documented for wireworms, the ubiquity of several genera suggests that at least some bacteria form beneficial or mutualistic associations with their host. *Pseudomonas* comprised 23.5% of the overall bacterial abundance and was the most represented genus for *Ae. mellillus* and late stage *L. californicus*. Members of this genus are noted animal and plant pathogens (Davies, 2002; Xin et al., 2018) and have potential as biocontrol agents (Chin‐A‐Woeng et al., 2000; Haas & Défago, 2005) and in bioremediation (Gilani et al., 2016; Huertas et al., 2006; O'Mahony et al., 2006; Saati‐Santamaría, Rivas, Kolařik, & García‐Fraile, 2021). It is often one of the most abundant genera identified in coleopterans (Chakraborty et al., 2020; Chamankar et al., 2023; Suárez‐Moo et al., 2020), including wireworms (Lacey et al., 2007; Zacharuk, 1973a; Zacharuk, 1973b; Zacharuk, 1974). For bark beetles, *Pseudomonas* symbionts have been shown to provide nutrients, detoxify their microenvironment and protect against pathogenic and antagonistic microorganisms (Saati‐Santamaría, Rivas, Kolařik, & Garcia‐Fraile, 2021; Saati‐Santamaría, Rivas, Kolařik, & García‐Fraile, 2021).

In addition to *Pseudomonas*, several other “core” bacterial genera were identified in high abundance in one or more wireworm species. The family Nocardiaceae (unresolved genus) accounted for 10.7% of the overall bacterial abundance and was particularly enriched in both *Hypnoidus* species. Members of this family are commonly found in soils and include eight phylogenetically closely related genera (Goodfellow, 2014; Stackebrandt et al., 1997). *Ehrlichia* (9.3%) and *Sphingoaurantiacus* (8.4%) were the next most abundant, largely due to their pervasiveness in *Dalopius* species. Little information is currently available for both genera, with the exception being the vertebrate pathogens of *Ehrlichia* (Aziz et al., 2022). *Acinetobacter* (5.8%) was present in relatively high abundance in both *L. californicus* and *H. abbreviatus*. This genus is prevalent in soils (Doughari et al., 2011) and frequently associated with the coleopteran bacteriome (Chakraborty et al., 2020; Chamankar et al., 2023; Dong & Yang, 2023). Given the lack of literature currently available for these and most other bacterial genera commonly harboured by wireworms, it is clear that considerable work is needed to better understand the symbiotic relationships between host and microbe beyond purely descriptive associations.

Although the microbial community composition differed extensively among wireworm species, few bacterial genera were species‐specific. This may suggest some similarities in microbial function among the different larvae. Network analyses provided insights into the associations of different bacteria harboured by wireworms, where some genera are more likely to be present concurrently across multiple species with similar patterns of change in relative abundance. We identified four networks, with each wireworm species represented in at least two of the networks. The exception was *Ae. mellillus*; similar to the species‐related reduced alpha diversity, its lack of microbial associations with other larvae is likely due to a unique reproductive strategy (Catton et al., 2021; Glen et al., 1943). While the parthenogenetic populations *H. bicolor* are also believed to occur, the population we sampled from is noted for containing both males and female beetles and are therefore likely sexual reproducing. The primary network of nine genera was consistent across sexual species and may be indicative of a core group of symbionts that are integral to the life history of these coleopterans (Gohl et al., 2022). The second largest network of three genera was specific to the *Hypnoidus* species, suggesting the co‐occurrence of some bacteria is associated with phylogenetic distance. Overall, the wireworm microbial communities appear to have some structural similarities that are consistent among diverse pest species; however, the interplays among the microorganisms and their influence (if any) on wireworm biology remains largely unresolved.

It is well established that the insect bacteriome is dynamic in nature, with a myriad of factors contributing to its plasticity (Engel & Moran, 2013; Muñoz‐Benavent et al., 2021; Paniagua Voirol et al., 2018). For coleopterans, the mechanisms by which their bacterial flora are acquired and maintained remain largely unknown. Suárez‐Moo et al. (2020) indicated that the dung beetle bacteriome was influenced by ontogeny. Cereal leaf beetle bacterial assemblages were also affected by development, larval host plant (i.e., diet) and sampling location (Wielkopolan et al., 2021). Other factors, such as farm management practices (Magagnoli et al., 2022), pesticide exposure (Giglio et al., 2021) and rearing conditions (Ibarra‐Juarez et al., 2018), can also impact beetle microbiota. The role of ontogeny in shaping the bacteriome of wireworms is intriguing, given that the larvae are long‐lived, ranging from 1 to 11 years (Andrews et al., 2020; Morales‐Rodriguez et al., 2014; Sandhi et al., 2020). Our insights into ontogeny were limited to early and late stage *L. californicus* and revealed considerable microbial differences, albeit not to the same extent as among species. In addition to ontogeny, diet and location are often cited as significant determinants of the insect bacteriome, and both were controlled to some degree in our study. Wireworms were captured pre‐seeding, meaning the overwintering larvae had nominal access to food sources outside the sterilised wheat used in bait traps. Consequently, the microbial composition of each species is presumably more indicative of a resident bacteriome rather than the choice of food substrate. However, it should be noted that many of the core bacterial genera harboured by wireworms have been isolated from soils, and in some cases, their presence could be transient reflecting the terricolous behaviours of wireworms. Wireworms were also collected from the same field, in a relatively small sampling area. While *H. abbreviatus* was collected from a nearby field, there did not appear to be unique signatures on the larval bacteriome indicative of a meaningful influence of location. In fact, the species showed more congruencies with *H. bicolor* and *L. californicus* than most other interspecies comparisons.

In conclusion, our study is the first to describe the bacteriomes of wireworms in the Canadian Prairies, encompassing some of the most important pest species. Moreover, all previous studies isolated bacteria from guts or other tissues of wireworms using selective media rather than sequencing the entire contents of the bacteriome. These coleopterans appear to have diverse and taxon‐rich bacterial flora, which differs considerably among larval species. However, a relatively small number of bacterial genera make up the bulk of their bacteriome, and there appears to be some community structuring consistent among diverse species. Both ontogeny and reproductive strategy had notable imprints on the bacterial composition, and it is likely that other factors (e.g., preparing to moult, host–microbe interactions) have contributed to bacteriome diversity that are not feasible to disentangle from our study but should be factored into future research. Subsequent studies are also needed to discern between transient and persistent (resident) bacteria harboured by the larvae. Measures of total bacterial abundance among wireworms were limited by the lack of genetic information (e.g., rDNA sequences) currently available for most species, which is needed for normalisation of microbial abundance to tissue DNA content. Much of the bacteria harboured by wireworms are poorly understood, highlighting the need for studies aimed at better understanding the symbiotic relationships between microbe and host.

## AUTHOR CONTRIBUTIONS

**Ivan Drahun:** Investigation; visualization; methodology; formal analysis; writing – review and editing. **Keagan Morrison:** Formal analysis; writing – review and editing. **Elise A. Poole:** Writing – review and editing; methodology; investigation. **Willem G. van Herk:** Conceptualization; writing – review and editing. **Bryan J. Cassone:** Conceptualization; investigation; funding acquisition; writing – original draft; formal analysis; project administration; supervision; data curation.

## FUNDING INFORMATION

This research was funded by the Natural Sciences and Engineering Research Council of Canada (NSERC) Discovery (RGPIN‐2016‐04335 and RGPIN‐2023‐04126) and Brandon University Research Committee (BURC) grants awarded to Bryan J. Cassone.

## CONFLICT OF INTEREST STATEMENT

The authors declare no conflicts of interest.

## Supporting information


**Figure S1.** Relative abundances of bacterial genera identified in five wireworm pest species. Each box plot represents a biological replicate for a given species. A total of 419 genera were identified across species, of which the 30 most represented are shown. For unknown genera, the lowest taxon level that could be resolved is provided.

**Table S1.** Sequencing statistics for each sequencing library. Approximately 38% of reads were omitted from formal analysis due to low quality, chimeric sequences and/or unable to be resolved to the OTU level.

**Table S2.** Differential abundance analysis at the genus‐level for each pairwise comparison among wireworm pest species.

**Table S3.** Habitat of the core bacterial genera (>1000 combined abundance).

## Data Availability

The raw sequence reads can be retrieved from the NCBI short sequence read archive (SRA) under the accession number PRJNA1098768.
