# Supplementary figures and images for: Characterisation of the bacteriomes harboured by major wireworm pest species in the Canadian Prairies
Source: Insect Mol Biol. 2024 Oct 9;34(1):203–17. doi: 10.1111/imb.12962 (PMC11705518; doi:10.1111/imb.12962)

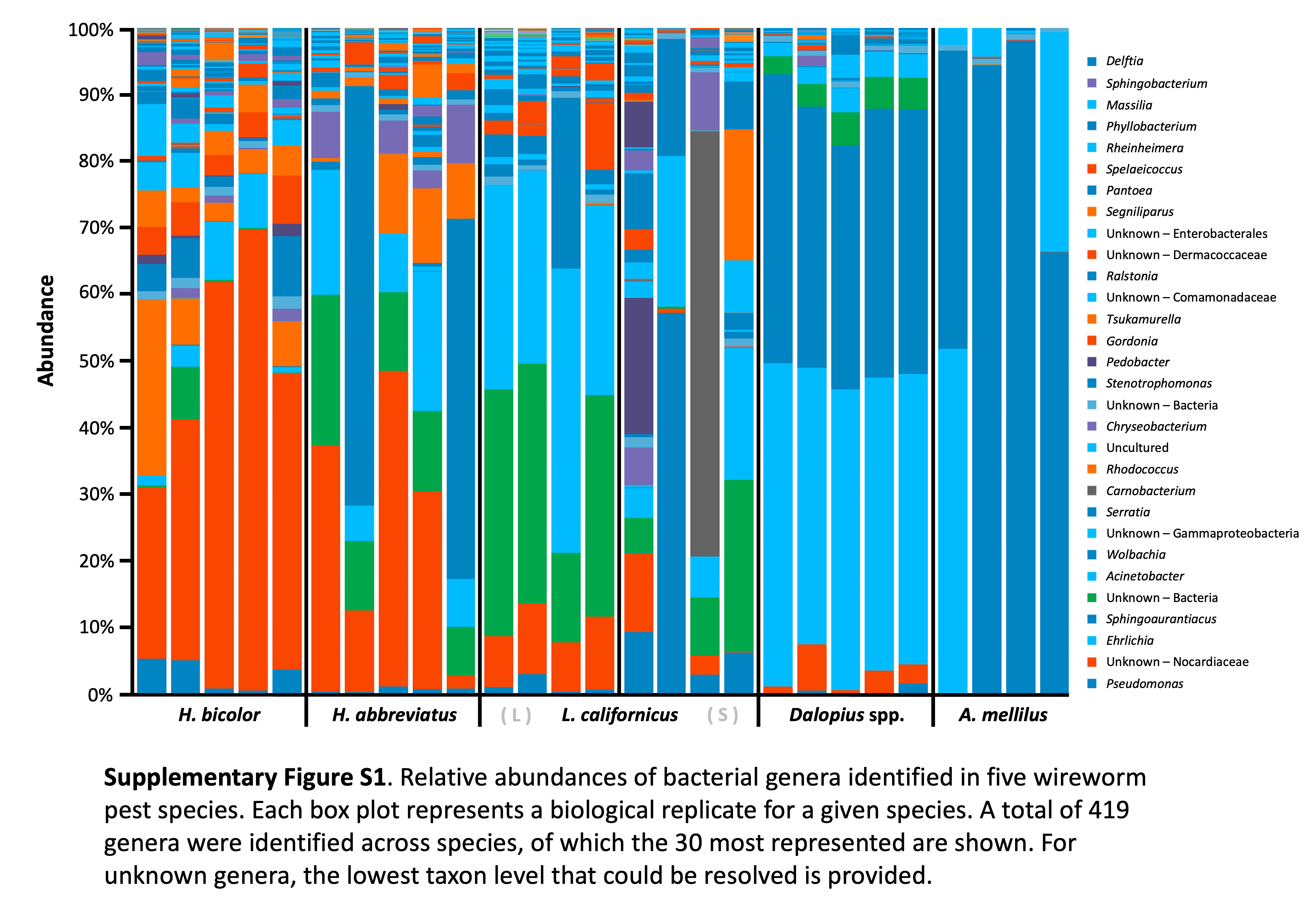

Supplement: Supplementary file 1 — Figure S1. Relative abundances of bacterial genera identified in five wireworm pest species. Each box plot represents a biological replicate for a given species. A total of 419 genera were identified across species, of which the 30 most represented are shown. For unknown genera, the lowest taxon level that could be resolved is provided. [file IMB-34-203-s002.png]
