# Supplementary material for: Characterisation of the bacteriomes harboured by major wireworm pest species in the Canadian Prairies
Source: Insect Mol Biol. 2024 Oct 9;34(1):203–17. doi: 10.1111/imb.12962 (PMC11705518; doi:10.1111/imb.12962)
Supplement: Supplementary file 2 — Table S1. Sequencing statistics for each sequencing library. Approximately 38% of reads were omitted from formal analysis due to low quality, chimeric sequences and/or unable to be resolved to the OTU level. [file IMB-34-203-s001.docx]

**Supplementary Table S1**. Sequencing statistics for each sequencing library. Approximately 38% of reads were omitted from formal analysis due to low quality, chimeric sequences, and/or unable to be resolved to the OTU level.

| **ID** | **Sample** | **Total number of reads** | **Filtered or chimeric reads** | **Reads in OTUs** |
| --- | --- | --- | --- | --- |
| *L. californicus* (S) | LC16 (paired, trimmed pairs) | 20,464 | 8,578 | 11,886 |
| *L. californicus* (S) | LC17 (paired, trimmed pairs) | 23,268 | 9,796 | 13,472 |
| *L. californicus* (S) | LC20 (paired, trimmed pairs) | 33,999 | 13,488 | 20,511 |
| *L. californicus* (S) | LC22 (paired, trimmed pairs) | 27,009 | 13,325 | 13,684 |
| *L. californicus* (L) | LC6 (paired, trimmed pairs) | 58,888 | 20,397 | 38,491 |
| *L. californicus* (L) | LC7 (paired, trimmed pairs) | 47,051 | 17,708 | 29,343 |
| *L. californicus* (L) | LC13 (paired, trimmed pairs) | 35,579 | 17,322 | 18,257 |
| *L. californicus* (L) | LC1 (paired, trimmed pairs) | 39,313 | 19,658 | 19,655 |
| *H. abbreviatus* | HA1 (paired, trimmed pairs) | 27,498 | 10,870 | 16,628 |
| *H. abbreviatus* | HA5 (paired, trimmed pairs) | 48,613 | 17,593 | 31,020 |
| *H. abbreviatus* | HA7 (paired, trimmed pairs) | 35,982 | 14,719 | 21,263 |
| *H. abbreviatus* | HA11 (paired, trimmed pairs) | 38,310 | 15,866 | 22,444 |
| *H. abbreviatus* | HA12 (paired, trimmed pairs) | 48,800 | 19,560 | 29,240 |
| *Dalopius* spp. | AG1 (paired, trimmed pairs) | 48,465 | 14,939 | 33,526 |
| *Dalopius* spp. | AG2 (paired, trimmed pairs) | 46,475 | 15,112 | 31,363 |
| *Dalopius* spp. | AG3 (paired, trimmed pairs) | 36,996 | 12,149 | 24,847 |
| *Dalopius* spp. | AG4 (paired, trimmed pairs) | 43,769 | 13,942 | 29,827 |
| *Dalopius* spp. | AG6 (paired, trimmed pairs) | 35,905 | 12,978 | 22,927 |
| *A. mellillus* | AE4 (paired, trimmed pairs) | 37,571 | 10,858 | 26,713 |
| *A. mellillus* | AE5 (paired, trimmed pairs) | 62,529 | 17,351 | 45,178 |
| *A. mellillus* | AE10 (paired, trimmed pairs) | 74,499 | 20,289 | 54,210 |
| *A. mellillus* | AE12 (paired, trimmed pairs) | 65,075 | 15,901 | 49,174 |
| *H. bicolor* | ED1 (paired, trimmed pairs) | 48,682 | 25,454 | 23,228 |
| *H. bicolor* | ED2 (paired, trimmed pairs) | 35,508 | 19,177 | 16,331 |
| *H. bicolor* | ED3 (paired, trimmed pairs) | 22,304 | 12,178 | 10,126 |
| *H. bicolor* | ED4 (paired, trimmed pairs) | 21,117 | 10,463 | 10,654 |
| *H. bicolor* | ED5 (paired, trimmed pairs) | 41,871 | 21,553 | 20,318 |
